# Research and Implementation of Indoor Positioning Algorithm Based on Bluetooth 5.1 AOA and AOD

**DOI:** 10.3390/s24144579

**Published:** 2024-07-15

**Authors:** Kun Xiao, Fuzhong Hao, Weijian Zhang, Nuannuan Li, Yintao Wang

**Affiliations:** 1 School of Computer Science and Engineering, University of Electronic Science and Technology, Chengdu 610056, China; 2 State Grid Henan Electric Power Company, Zhengzhou 450018, China; cnhfzs@sina.com.cn (F.H.); zhangweijian@ha.sgcc.com.cn (W.Z.); 3 State Grid Henan Electric Power Research Institute, Zhengzhou 450000, China; 18339231859@163.com

**Keywords:** indoor positioning, Bluetooth AOA/AOD, DOA estimation algorithm, least squares algorithm, anti-multipath interference algorithm

## Abstract

With the addition of Bluetooth AOA/AOD direction-finding capabilities in the Bluetooth 5.1 protocol and the introduction of antenna array technology into the Bluetooth platform to further enhance positioning accuracy, Bluetooth has gradually become a research hotspot in the field of indoor positioning due to its standard protocol specifications, rich application ecosystem, and outstanding advantages such as low power consumption and low cost compared to other indoor positioning technologies. However, current indoor positioning based on Bluetooth AOA/AOD suffers from overly simplistic core algorithm implementations. When facing different application scenarios, the standalone AOA or AOD algorithms exhibit weak applicability, and they also encounter challenges such as poor positioning accuracy, insufficient real-time performance, and significant effects of multipath propagation. These existing problems and deficiencies render Bluetooth lacking an efficient implementation solution for indoor positioning. Therefore, this paper proposes a study on Bluetooth AOA and AOD indoor positioning algorithms. Through an analysis of the principles of Bluetooth’s newly added direction-finding functionality and combined with research on array signal DOA estimation algorithms, the paper ultimately integrates the least squares algorithm to optimize positioning errors in terms of accuracy and incorporates an anti-multipath interference algorithm to address the impacts of multipath effects in different scenarios. Experimental testing demonstrates that the indoor positioning algorithms applicable to Bluetooth AOA and AOD can effectively mitigate accuracy errors and overcome multipath effects, exhibiting strong applicability and significant improvements in real-time performance.

## 1. Introduction

With the rapid development of wireless communication technology and the widespread application of Internet of Things (IOT) technology, location-based services have greatly promoted the development of social production and life. In outdoor environments, the development of satellite positioning technologies such as the Global Positioning System (GPS), the BeiDou Navigation Satellite System, and GLONASS has matured significantly. Consequently, research has gradually shifted towards indoor positioning technologies [1]. Currently, mainstream indoor positioning technologies include ultra-wideband (UWB), WiFi, radio-frequency identification (RFID), and Bluetooth Low Energy, among others. Among these, Bluetooth Low Energy technology, represented by its standardized protocol specifications, combined with its rich ecosystem, low power consumption, and cost-effectiveness [2], has gradually become the focus of current research in indoor positioning.

The Bluetooth Technology Alliance added a direction-finding function to the Bluetooth 5.1 protocol specification, which includes two methods: angle of arrival (AOA) and angle of emission (AOD). Currently, the direction-finding function of Bluetooth is still in the early stages of development, and further research and improvement are needed based on the practical application of its technology. Some proposed AOA/AOD estimation methods, such as the indoor positioning system that integrates inertial navigation and Bluetooth AOA, use auxiliary devices such as gyroscopes and accelerometers combined with Bluetooth to form a high-precision AOA positioning system. However, when it is applied in different scenarios, the applicability of the software algorithm is poor and it is difficult to solve prominent environmental problems; while others, such as the three-dimensional indoor positioning system that integrates Bluetooth AOA/AOD and ultra-wideband, use an ultra-wideband base station in combination with a Bluetooth base station and add a filtering algorithm to complete indoor three-dimensional positioning. There are still many deficiencies in the research and optimization of the algorithm part. The algorithm depends on the choice of hardware and has poor universality [3,4]. In the implementation of positioning algorithms based on Bluetooth 5.1, there are still many problems and defects at this stage, including the improvement of positioning accuracy, the influence of multipath effects in different environments, and the weak applicability of the algorithms of AOA and AOD. Therefore, this paper proposes a study on DOA estimation algorithms suitable for two Bluetooth direction-finding methods, and further integrates the positioning algorithm’s accuracy and a solution to multipath interference in different environments to achieve high-precision output of the entire positioning algorithm.

The structure of this paper is as follows: Firstly, by studying the principles of Bluetooth direction-finding, the implementation process, data information, and positioning principles of direction-finding technology are analyzed. Secondly, based on the research findings, a further study is conducted to analyze direction-of-arrival (DOA) estimation algorithms suitable for both Bluetooth AOA and AOD methods, and a more suitable algorithm is selected for further positioning research. Finally, according to the selected algorithm and the issues encountered in practical applications, optimization algorithms are adopted to further design and experimentally test the positioning algorithm. The experimental results are analyzed, research directions are summarized, and the provided application value is evaluated, aiming to provide efficient implementation solutions for more researchers.

We summarize the contributions of our work as follows:

(1) We researched and analyzed the implementation principles, process, and data information used in the positioning process of the newly added direction-finding feature in Bluetooth 5.1. Furthermore, we summarized the entire positioning process to provide more detailed reference for other researchers.

(2) We researched and analyzed direction-of-arrival (DOA) estimation algorithms suitable for Bluetooth AOA and AOD. Through testing and data comparison analysis, we selected DOA estimation algorithms with stronger applicability for further research and improvement.

(3) Based on the research outcomes of the algorithms and considering the existing issues and deficiencies in practical applications, we optimized the algorithms by employing the least squares and anti-multipath interference algorithms, resulting in the design of a Bluetooth indoor positioning algorithm with strong versatility.

## 2. Research on Bluetooth AOA/AOD Technology

### 2.1. Principles of CTE Signals

**Figure 1:**
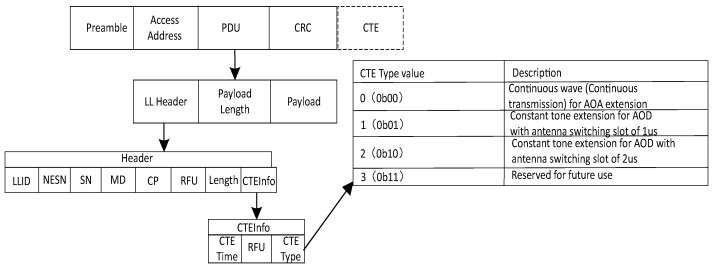
CTE parameter configuration information.

The Bluetooth 5.1 specification introduces direction-finding capabilities, which essentially involve adding a portion of CTE (Constant Tone Extension) fields to the end of Bluetooth data packets. The overall parameter information of CTE is illustrated in Figure 1 CTE consists of a segment of continuous binary symbols “1”, the quantity of which can be configured in the Bluetooth protocol stack. With Bluetooth 5.1 hardware, the transmitter sends CTE at a stable frequency. As it consists of a continuous sequence of characters “1”, the receiver can sample this stable frequency [5,6]. Finally, the sampled data are saved in I/Q format for further use in direction-finding functionality.

The CTE (Constant Tone Extension) can be sent in both connection mode and broadcast mode [7]. It is appended after the CRC (cyclic redundancy check) without affecting the original data content. The configuration of CTE can be specified in the header of the link layer data packet PDU (protocol data unit), including settings for CTE type and duration, as shown in Figure 1. In the CTEInfo field, CTETime represents the duration of CTE in units of 8 microseconds, ranging from 2 to 20. The CTEType field indicates the type of CTE and the slot size for antenna switching, with other values reserved for future use [8].

**Figure 2:**
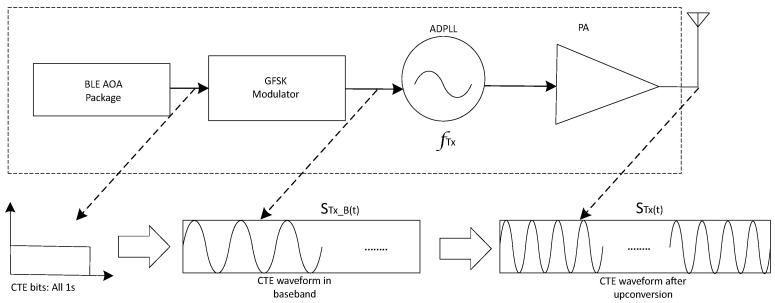
Diagram of CTE transmission-end principle.

Taking AOA mode as an example, the principle of the Bluetooth direction-finding transmitter is shown in Figure 2, where the bits of CTE pass through a Gaussian frequency-shift keying (GFSK) modulator [9], with its baseband waveform as follows:

(1) $$S_{{\mathrm{TX}}_{-}B}\left(t\right)=A_{{\mathrm{TX}}_{-}B}\cos\left(2\pi \int_{dev}t+\theta_{{\mathrm{TX}}_{-}B}\right)$$

Here, $\theta_{{\mathrm{TX}}_{-}B}$ represents the initial phase and $\int_{dev}$ represents the frequency deviation.

The baseband waveform is up-converted to the Bluetooth channel frequency, then amplified by a power amplifier for transmission. The transmitted signal waveform of the CTE bits is

(2) $$S_{TX}\left(t\right)=A_{TX}\cos\left(2\pi \int_{dev}t+2\pi \int_{TX}t+\theta_{TX}\right)$$

where $\int_{TX}$ represents the Bluetooth channel frequency, and $\theta_{TX}$ represents the initial phase at antenna transmission.

**Figure 3:**
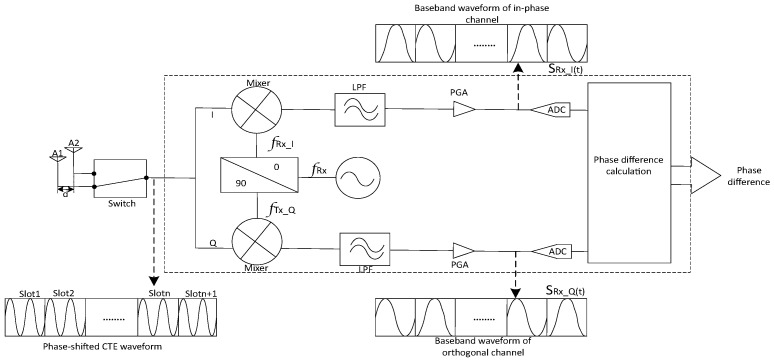
Diagram of CTE receiver-end principle.

As shown in Figure 3, at the Bluetooth device receiver, the Bluetooth receiver switches through the array elements one by one using a time-division switching mechanism. Taking the example of a receiver with two antennas, the signals received by the antennas are

(3) $$\begin{array}{c} S_{{\mathrm{RX}}_{-}S}\left(t\right)\\ =\left\{\begin{array}{c} A_{\mathrm{RX}}\cos\left(2\pi \left(\int_{\mathrm{dev}}+\int_{\mathrm{TX}}\right)\left(t-\frac{d_{1}}{c}\right)+\theta_{RX}\right)+n\left(t\right),t\in t_{\mathrm{slot}\;}\left[2n,2n+1\right)\\ A_{RX}\cos\left(2\pi \left(\int_{dev}+\int_{TX}\right)\left(t-\frac{d_{2}}{c}\right)+\theta_{RX}\right)+n\left(t\right),t\in t_{\mathrm{slot}\;}\left[2n+1,2n+2\right) \end{array}\right.\\ =\left\{\begin{array}{c} A_{RX}\cos\left(2\pi \left(\int_{dev}+\int_{TX}\right)t+\theta_{RX_{-}1}\right)+n\left(t\right),t\in t_{\mathrm{slot}\;}\left[2n,2n+1\right)\\ A_{RX}\cos\left(2\pi \left(\int_{dev}+\int_{TX}\right)t+\theta_{RX_{-}1}+\theta_{d}\right)+n\left(t\right),t\in t_{\mathrm{slot}\;}\left[2n+1,2n+2\right) \end{array}\right. \end{array}$$

where $d_{1}$ and $d_{2}$ represent the distances on the signal propagation path between the transmitting antenna and the two receiving antennas, respectively, n(t) represents the received noise, and $t_{\mathrm{slot}\;}$ represents the duration of each antenna sampling slot.

(4) $$\theta_{d}=2\pi \left(\int_{dev}+\int_{TX}\right)\frac{d_{1}-d_{2}}{c}=2\pi \frac{d}{\lambda_{TX}}$$

where d represents the distance between the two receiving antennas on the signal propagation path, and $\lambda_{TX}$ is the wavelength of the transmitted signal.

At the receiver end, down-conversion is performed first, followed by orthogonal sampling. The outputs of the in-phase channel and the quadrature channel are

(5) $$\begin{array}{cc} \; & S_{RX_{I}}\left(t\right)\\ \; & \;=\left\{\begin{array}{c} A_{IQ}\cos\left(2\pi \left(\int_{dev}+\Delta \int_{car}\right)t+\theta_{RXIQ_{-1}}\right)+n_{I}\left(t\right),t\in t_{\mathrm{slot}\;}\left[2n,2n+1\right)\\ A_{IQ}\cos\left(2\pi \left(\int_{dev}+\Delta \int_{\mathrm{car}\;}\right)t+\theta_{RXIQ_{-}1}+\theta_{d}\right)+n_{I}\left(t\right),t\in t_{\mathrm{slot}\;}\left[2n+1,2n+2\right) \end{array}\right. \end{array}$$

(6) $$\begin{array}{c} S_{RX_{Q}}\left(t\right)\\ =\left\{\begin{array}{c} A_{IQ}\sin\left(2\pi \left(\int_{dev}+\Delta \int_{\mathrm{car}\;}\right)t+\theta_{RXIQ\_1}\right)+n_{Q}\left(t\right),t\epsilon t_{\mathrm{slot}\;}\left[2n,2n+1\right)\\ A_{IQ}\sin\left(2\pi \left(\int_{\mathrm{dev}\;}+\Delta \int_{\mathrm{car}\;}\right)t+\theta_{RXIQ_{-}1}+\theta_{d}\right)+n_{Q}\left(t\right),t\epsilon t_{\mathrm{slot}\;}\left[2n+1,2n+2\right) \end{array}\right. \end{array}$$

where $\Delta \int_{\mathrm{car}\;}$ represents the carrier frequency offset between the transmitter and the receiver, i.e., $\Delta \int_{\mathrm{car}\;}=\int_{TX}-\int_{RX}\cdot n_{I}\left(t\right)$ and $n_{Q}\left(t\right)$, respectively, represent the noise received in the in-phase channel and the quadrature channel.

### 2.2. Angle Calculation and Coordinate Transformation

**Figure 4:**
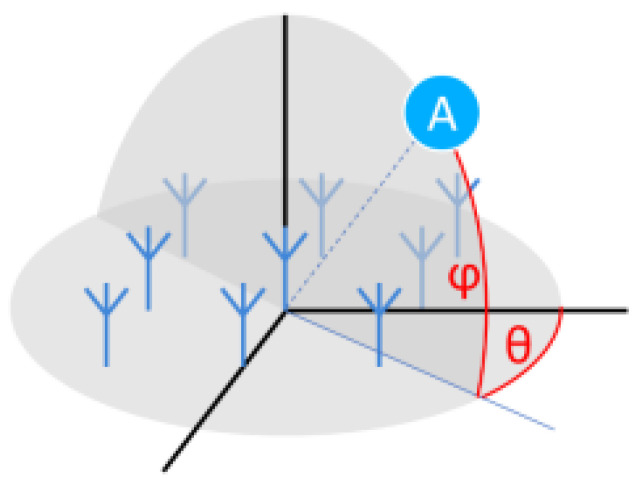
Angle direction of two-dimensional antenna array.

A one-dimensional antenna can effectively measure the azimuth angle θ, while a two-dimensional antenna can further obtain the elevation angle φ of the target in three-dimensional space, as shown in Figure 4. Based on the two-dimensional antenna array, the direction angle and elevation angle of target A can be accurately measured [10], thereby preliminarily determining the position information of target A in three-dimensional space.

**Figure 5:**
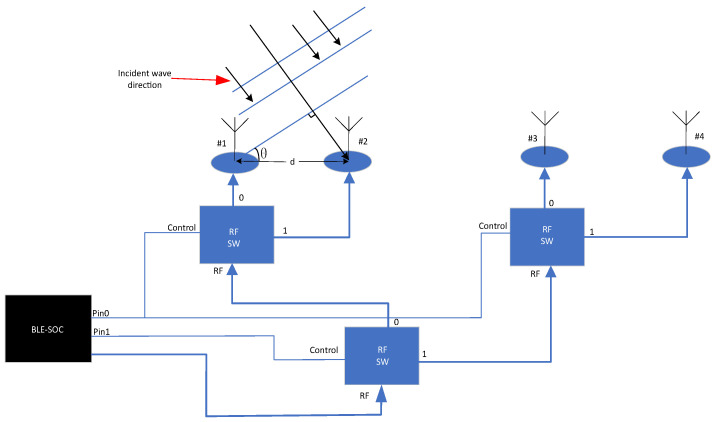
Principle of angle calculation.

From Figure 4, it is evident that to obtain the spatial position information of the target, the most crucial aspect is acquiring the corresponding angle information. With this information, the specific position of the target can be calculated further [11]. As shown in Figure 5, the receiving-end antenna controlled by the Bluetooth chip is depicted. When the antenna receives the incident wave signal, since the wavelength λ of 2.4 GHz is approximately 0.125 m, and the distance d between the antennas is known, along with the phase difference ϕ of the signal, the angle of incidence θ relative to the receiving end of the target can be calculated using Formula (7). For a two-dimensional antenna [12], the same principle can also be used to measure the elevation angle φ of the target relative to the receiving end.

(7) $$d\cos\theta =\frac{\phi \lambda }{2\pi }$$

**Figure 6:**
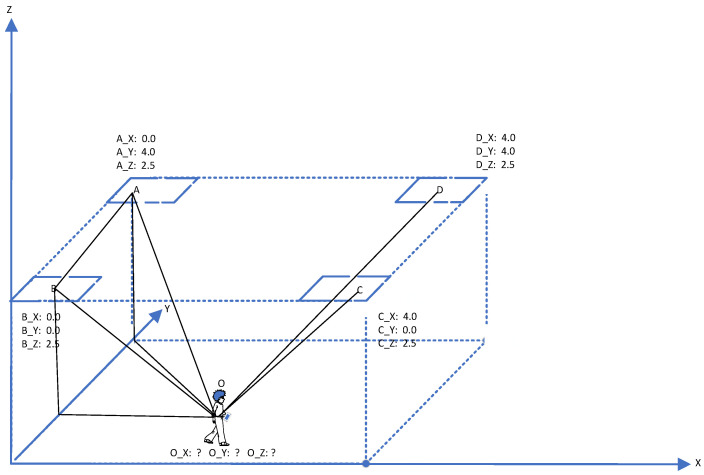
Principle of coordinate calculation.

Once accurate angles relative to the receiving end are obtained, by combining multiple (≥2) receiving-end devices and utilizing the triangular relationship formed by the angle information and the devices, the target’s position coordinates can be calculated using the triangulation method, as shown in Figure 6, where A, B, C, and D represent four two-dimensional antenna arrays positioned on the XYZ coordinate axis in three-dimensional space. The coordinates of each antenna array are known [13,14], and the coverage area formed fully encompasses the test environment. However, the coordinates of point O are unknown, indicating the need to determine the position coordinates of the target O. As seen in the figure, any two receiving-end devices can form a triangular relationship with the target to be measured, and ≥2 devices can form multiple triangular relationships to achieve more accurate and higher-precision positioning.

As shown in Figure 6, the two-dimensional antenna arrays A and B are taken as examples to obtain the position coordinates of target O, assuming the coordinates of A are (X1, Y1) and the coordinates of B are (X2, Y2), and the angles ∠ABO and ∠BAO can be obtained based on the antenna arrays. Moreover, by utilizing the triangular relationship formed by the two receiving-end devices and the target, the coordinates (X, Y) of target O can be determined using the triangulation method. Similarly, for three-dimensional coordinates, target information can be obtained using the same principle.

(8) $$X=-\frac{\left(Y2-X2\tan∠\mathrm{BAO})-(Y1-X1\tan∠\mathrm{ABO}\right)}{\tan∠\mathrm{BAO}-\tan∠\mathrm{ABO}}$$

(9) $$Y=-\frac{\left(X2-Y2\cot∠\mathrm{BAO})-(X1-Y1\cot∠\mathrm{ABO}\right)}{\cot∠\mathrm{BAO}-\cot∠\mathrm{ABO}}$$

## 3. Research on Direction-of-Arrival (DOA) Estimation Algorithms

### 3.1. ESPRIT Algorithm

The ESPRIT algorithm is based on the subspace rotation-invariant technique, which eliminates the need for a full-space search, reducing computational complexity. Due to its superiority in parameter estimation and other aspects, the algorithm has been widely applied in recent years [15,16]. The following section provides a detailed introduction to its core implementation in DOA estimation.

Step 1: Divide the two-dimensional antenna array into several rectangular sub-arrays of overlapping sizes, calculate the covariance matrix of the received signals for each sub-array, and further, obtain the average value $\bar{R}$.

(10) $$\bar{R}=\frac{1}{M_{s}N_{s}}\sum_{m=1}^{M_{s}}\sum_{n=1}^{N_{s}}R_{mn}$$

Step 2: Perform eigendecomposition on the average value $\bar{R}$ to obtain the signal subspace $E_{s}$, and divide it into blocks. Obtain ${\hat{U}}_{K}$ from the eigenvalues of $E_{x}+E_{y}$, then reconstruct $E_{s}$. Similarly, obtain ${\hat{V}}_{K}$ using a similar method.

As shown in the following formula, $E_{s}$ represents the signal subspace matrix obtained by signal eigenvalue decomposition, $E_{s}1$ and $E_{s}2$ represent the last M rows and the first M rows of the matrix $E_{s}$, respectively, k represents the signal target in the space (k = 1, 2, …, k), M represents the number of antenna array elements (M = 1, 2 …, M), and $\gamma_{k}$ represents the Kth eigenvalue of the matrix $E_{x}+E_{y}$.

(11) $$\begin{array}{c} E_{s}=\left[\begin{array}{c} E_{x}\\ E_{s1} \end{array}\right]=\left[\begin{array}{c} E_{s2}\\ E_{y} \end{array}\right]\\ {\hat{U}}_{k}=-\mathrm{angle}\left(\gamma_{k}\right)\lambda /2\pi d\\ {\hat{V}}_{k}=\cos\theta_{k}\sin\varphi_{k} \end{array}$$

Step 3: Calculate ${\hat{\theta }}_{k}$ and ${\hat{\varphi }}_{k}$ using the equation.

(12) $$\left\{\begin{array}{c} {\hat{\theta }}_{k}=\tan^{-1}\left({\hat{U}}_{k}/{\hat{V}}_{k}\right)\\ {\hat{\varphi }}_{k}=\sin^{-1}\sqrt{{\hat{U}}_{k}^{2}+{\hat{V}}_{k}^{2}} \end{array}\right.$$

According to the research on the principle and implementation process of the algorithm, a verification test and analysis of the data simulation are carried out. The Matlab software is used to set the antenna element spacing, number of elements, and signal-to-noise ratio to simulate the signal. At the same time, the simulated signal is sampled 1000 times in combination with the ESPRIT algorithm. Finally, the solution is obtained to obtain the corresponding azimuth and elevation angle results, and the accuracy and calculation time are evaluated. The results are recorded in Table 1. According to the results, it can be analyzed that the algorithm is lacking in azimuth accuracy and the solution time performance is not very good, which needs to be improved.

### 3.2. Capon Algorithm

The Capon algorithm has the advantage of automatic parameter pairing in two-dimensional DOA estimation, but its complexity is high due to the spatial spectrum. Therefore, in practical applications, improved algorithms with dimensionality reduction are commonly used. Similar to other DOA estimation algorithms, its implementation principle is also based on spectrum peak search, where the two angles corresponding to the spectrum peaks are the desired azimuth and elevation angles [17,18]. The following introduces the core implementation process of the dimensionality reduction-based algorithm:

Step 1: Calculate the covariance matrix of the received signals.

(13) $$\hat{R_{x}}=\frac{1}{\;L}\sum_{I=1}^{L}X\left(t_{1}\right)X^{H}\left(t_{1}\right)$$

Step 2: Perform a global search to find the K largest peaks in the (1,1) element of $Q{\left(U\right)}^{-1}$, obtaining estimated values for $U_{k}\left(K=1,\ldots ,K\right)$, Calculate ${\hat{a}}_{x}\left(V_{k}\right)$ using the following equation, and then, use least squares to obtain estimated values for $V_{k}\left(K=1,\ldots ,K\right)$. Where L is the number of samples, $Q{\left(U\right)}^{-1}$ is the inverse matrix of matrix U, and $e_{1}$ is the transposed matrix representing the row matrix.

(14) $${\hat{a}}_{x}\left(V_{k}\right)=\frac{Q{\left(U\right)}^{-1}e_{1}}{e_{1}^{H}Q{\left(U\right)}^{-1}e_{1}}$$

Step 3: Finally, calculate ${\hat{\theta }}_{k}$ and ${\hat{\varphi }}_{k}$ using the following formula.

(15) $$\left\{\begin{array}{c} {\hat{\theta }}_{k}=\tan^{-1}\left({\hat{U}}_{k}/{\hat{V}}_{k}\right)\\ {\hat{\varphi }}_{k}=\sin^{-1}\sqrt{{\hat{U}}_{k}^{2}+{\hat{V}}_{k}^{2}} \end{array}\right.$$

According to the research on the principle and implementation process of the algorithm, a verification test and analysis of the data simulation are carried out. The Matlab software is used to set the antenna element spacing, number of elements, and signal-to-noise ratio to simulate the signal. At the same time, the Capon algorithm is combined to sample the simulated signal 1000 times, and finally, the solution is obtained to obtain the corresponding azimuth and elevation angle results, and the accuracy and calculation time are evaluated. The results are recorded in Table 2. According to the results, it can be analyzed that the algorithm is lacking in accuracy at two angles, and is not as good as the ESPRIT algorithm in terms of solution time.

### 3.3. MUSIC Algorithm

The MUSIC algorithm is the most commonly used method for two-dimensional DOA estimation. Compared to the other algorithms, it is relatively simple in terms of principles and implementation steps. However, it has the advantage of quick verification or development, and there are more methods for improvement and optimization [19,20]. Nevertheless, it also faces the issue of high computational complexity, which can be mitigated by using the improved cascaded MUSIC algorithm. The core implementation process is as follows.

Step 1: Utilize the received signals to obtain an estimated covariance matrix.

(16) $$\hat{R_{x}}=\frac{1}{\;L}\sum_{I=1}^{L}X\left(t_{1}\right)X^{H}\left(t_{1}\right)$$

Step 2: Perform eigendecomposition on the estimated covariance matrix ${\hat{R}}_{x}$ to obtain $E_{s}$ and $E_{n}$. Obtain ${\hat{U}}_{K^{ini}}$ from the eigenvalues of $E_{x}+E_{y}$, and apply the one-dimensional MUSIC principle to obtain ${\hat{V}}_{k}$,

(17) $$\begin{array}{cc} {\hat{u}}_{k}=\arg\max_{u\in \left[{\hat{U}}_{k^{ini}}-\Delta ,{\hat{U}}_{k^{ini}}+\Delta \right]}\frac{1}{{\left[a_{1}\left(u\right)*a_{2}\left({\hat{V}}_{k}\right)\right]}^{H}E_{n}E_{n}^{H}\left[a_{1}\left(u\right)*a_{2}\left({\hat{V}}_{k}\right)\right]} & k=1,2,\cdots ,K\\ {\hat{v}}_{k}=\arg\max_{v\in [-1,+1]}\frac{1}{{\left[a_{1}\left({\hat{U}}_{k^{ini}}\right)*a_{2}\left(v\right)\right]}^{H}E_{n}E_{n}^{H}\left[a_{1}\left({\hat{U}}_{k^{ini}}\right)*a_{2}\left(v\right)\right]} & k=1,2,\cdots ,K \end{array}$$

Step 3: Finally, ${\hat{\theta }}_{k}$ and ${\hat{\varphi }}_{k}$ can be calculated using the following equation.

(18) $$\left\{\begin{array}{c} {\hat{\theta }}_{k}=\tan^{-1}\left({\hat{U}}_{k}/{\hat{V}}_{k}\right)\\ {\hat{\varphi }}_{k}=\sin^{-1}\sqrt{{{\hat{U}}_{k}}^{2}+{{\hat{V}}_{k}}^{2}} \end{array}\right.$$

**Figure 7:**
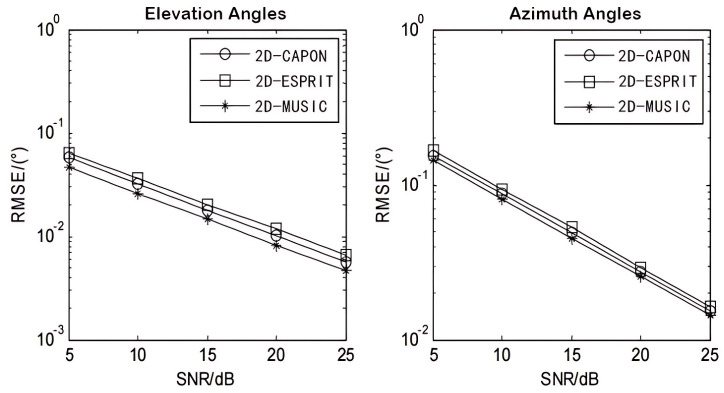
Comparison of algorithms’ angle estimation performance.

According to the research on the principle and implementation process of the algorithm, a verification test and analysis of the data simulation are carried out, and the results are recorded in Table 3. The three algorithms studied are compared and performance analysis is carried out. As shown in Figure 7, whether it is the elevation estimation diagram on the left or the azimuth estimation diagram on the right, when the signal-to-noise ratio data (SNR) gradually increases, the error (RMSE) of the three algorithms gradually decreases, and the performance of the MUSIC algorithm is outstanding. Compared with the Capon and ESPRIT algorithms, the performance is better. It can be seen that the MUSIC algorithm has certain advantages in performance. The subsequent design based on this algorithm will realize further positioning algorithm research.

## 4. Analysis and Design of Bluetooth AOA/AOD Indoor Positioning Algorithm

In indoor positioning, signals emitted by signal sources undergo distortion during propagation due to environmental factors, leading to deviations in the received signals and resulting in inaccurate positioning results. Unlike outdoor open environments, indoor environments are complex and diverse, exerting a significant impact on positioning. Particularly, indoor multipath effects manifest as wireless signals undergoing multiple reflections, scattering, and diffraction before reaching the receiving antenna through multiple paths during propagation. This phenomenon significantly degrades the accuracy of indoor positioning. Therefore, analyzing and mitigating indoor multipath propagation is indispensable.

**Figure 8:**
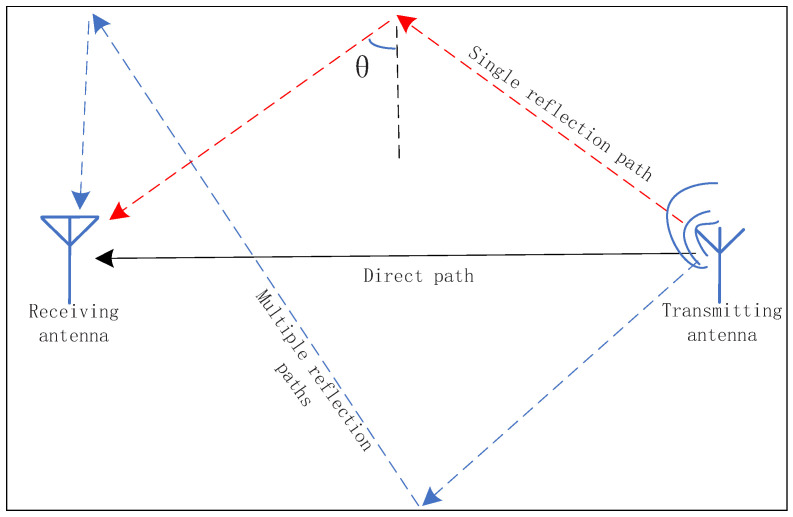
Specular reflection multipath signal model.

The multipath signal model with specular reflection is illustrated in Figure 8. Apart from the direct signal, indoor environments also involve reflected signals from different paths, which may vary in the number of reflections. Taking the example of a single reflected signal path shown in the figure, with an incident angle of θ and a straight-line distance between the transmitting and receiving antennas of D, the delay of the reflected signal compared to the direct signal after one reflection is

(19) $$\Delta L=D\left(\frac{1}{\sin\theta }-1\right)$$

**Figure 9:**
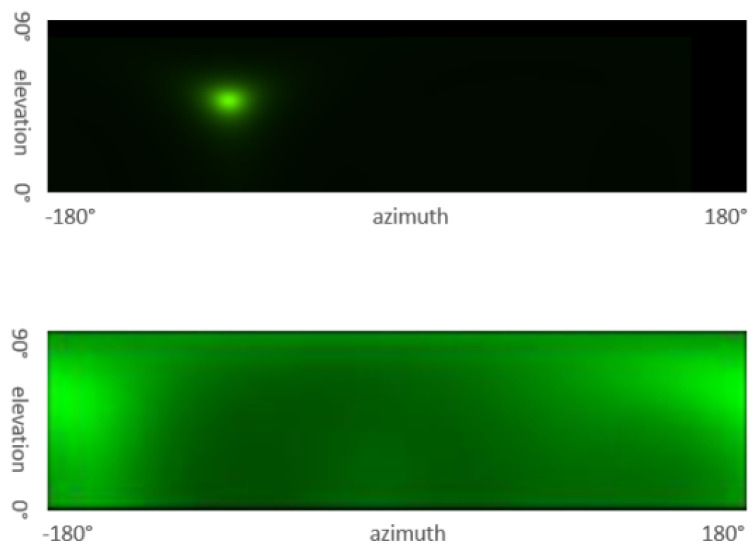
Pseudo-spectrum comparison in ideal and real environments.

The MUSIC algorithm has good resolution and high accuracy in a good signal-to-noise ratio environment. When the BLE signal strength is not particularly prominent, especially in an indoor environment, the multipath effect distorts the pseudo-spectrum, resulting in the maximum value of the angle estimation appearing at the wrong position. A spectrum analyzer is used in an experimental environment without interference and in an open space to obtain the spectrum distribution diagram of the Bluetooth signal in an ideal situation. At the same time, a spectrum analyzer is also used in a real environment to collect the spectrum distribution diagram of the Bluetooth signal in actual applications. Figure 9 compares the pseudo-spectra in the ideal situation and the real situation. It shows that the algorithm should increase the processing design of the multipath effect in actual applications, and the algorithm can be used to further improve the range accuracy performance.

### 4.1. Algorithm Improvement Design

#### 4.1.1. Least Squares Algorithm

Utilizing mathematical optimization techniques, finding the best function match for the data by minimizing the sum of squared errors, establishing measurement equations based on the geometric principles of positioning methods, and if necessary, linearizing combined nonlinear equations to obtain a set of joint equations, thus enabling a more accurate determination of the target’s position, can effectively improve the positioning accuracy of the algorithm. The following is the design and implementation process.

(20) Y=AX

where Y typically represents an nx1-dimensional known vector expressed as a function of measurement values, X is an mx1-dimensional position vector consisting of the coordinates of the tags’ locations, and A is an nxm-dimensional measurement matrix representing the relationship between measurement values and tag positions. Due to various errors, the above system of equations usually has no solution. Introducing an error vector ε, we have

(21) ε=AX−Y

The problem can be transformed into finding X that minimizes the sum of squared residuals:

(22) $$\int \left(X\right)={\left(AX-Y\right)}^{T}\left(AX-Y\right)$$

Taking the derivative of ∫(X) and setting it equal to zero, we have

(23) $$\frac{\partial \int (X)}{dX}=2A^{T}AX-2AY=0$$

If m = n and A is non-singular, then $X=A^{-1}Y$; if n>m, that is, the number of equations is greater than the number of unknowns, then the least squares solution is obtained. When $A^{T}A$ is non-singular, the least squares solution is

(24) $$\hat{X}={\left(A^{T}A\right)}^{-1}A^{T}Y$$

In practical applications, it is often necessary to use the weighted least squares algorithm to address issues related to varying precision in different measurement values. Based on different measurement methods or factors such as varying signal quality at different measurement nodes, a weighting matrix W is formulated. With the above equation, we have $\int_{W}\left(X\right)={\left(AX-Y\right)}^{T}W\left(AX-Y\right)$. Using the same method for differentiation, we obtain the weighted least squares solution:

(25) $$\hat{X}w={\left(A^{T}WA\right)}^{-1}A^{T}WY$$

**Figure 10:**
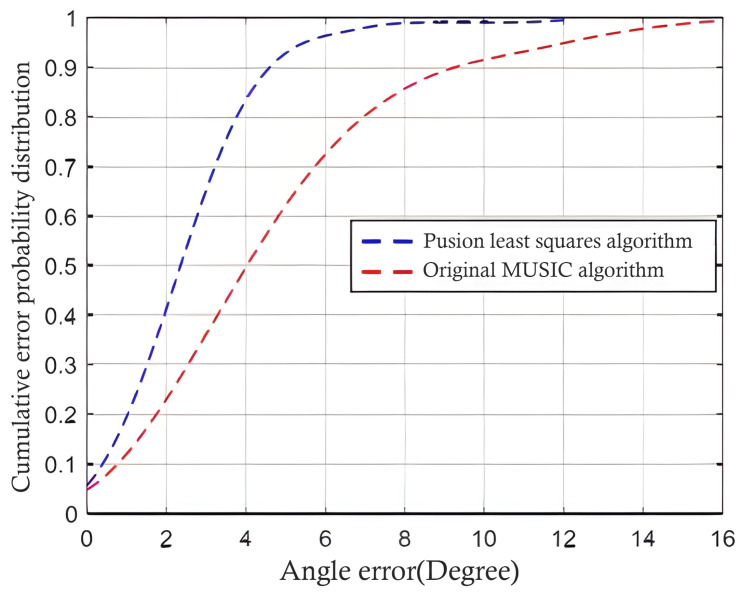
Comparison of estimation errors for improved algorithm.

Taking the base station A in Figure 6 as an example, with the coordinates of the base station being $\left(x_{i},y_{i}\right)$, and the coordinates of the target O being (x, y), we have

(26) $$\begin{array}{c} {\left(x-x_{i}\right)}^{2}+{\left(y-y_{i}\right)}^{2}=d_{i}^{2}\\ \tan\left(\theta_{i}\right)=\frac{y-y_{i}}{x-x_{i}} \end{array}$$

where i = 0, 1, 2, …, N−1, according to the above equation group (26), by linearizing the first equation group by subtracting the first equation in turn, the final linear equation group can be obtained as follows (27), and combined with Formula (24) and Formula (25), the required target (x, y) information can be finally solved. By integrating the overall least squares algorithm into the MUSIC algorithm in DOA estimation, the accuracy of the algorithm in indoor positioning can be further improved. The initial MUSIC algorithm and the improved algorithm are sampled and calculated under the same simulation signal, and the simulation method is the same as in Table 1. At the same time, the angle errors of the two algorithms are recorded to form an error probability distribution curve, as shown in Figure 10. It can be clearly seen that the positioning accuracy based on the improved algorithm has been significantly improved.

(27) $$A=\left[\begin{array}{cc} 2\left(x_{1}-x_{0}\right) & 2\left(y_{1}-y_{0}\right)\\ \tan\theta_{0} & -1\\ \tan\theta_{N-1} & -1 \end{array}\right],\;Y=\left[\begin{array}{c} x_{1}^{2}-x_{0}^{2}+y_{1}^{2}-y_{0}^{2}+d_{0}^{2}-d_{1}^{2}\\ \ldots \\ x_{0}\tan\theta_{N-1}-y_{0}\\ x_{N-1}\tan\theta_{N-1}-y_{N-1} \end{array}\right]$$

#### 4.1.2. Anti-Multipath Interference Algorithm

The spectral peak search algorithm can be used to obtain an angle sequence containing effective signals and some multipath signals: $\;{\mathrm{Angle}}^{{AP}_{1}}=\left\{\left(\theta_{h_{1}}^{AP_{1}},\theta_{v_{1}}^{AP_{1}}\right),\ldots ,\left(\varphi_{hk}^{AP_{1}},\varphi_{vk}^{AP_{1}}\right)\right\}$. The information in this sequence is sorted according to the probability of the spatial spectrum, and the quantity can be set considering the richness of the indoor multipath and the computational speed of the positioning backend device. Then, for each base station, the first value in the angle sequence is selected, i.e., the path with the highest probability, to generate the corresponding matrices A and B along with their weight matrix W. The positioning result and residual error $\;\mathrm{error}\;=\mathrm{mean}\left(\mathrm{abs}\left(A{\hat{P}}_{B}^{W}-B\right)\right)$ are calculated. If the residual error is less than the threshold, the current calculated position is taken as the final positioning result. Otherwise, the remaining angle sequences for each base station are traversed until a solution with a residual error less than the threshold is found. If after traversing all angle sequences the residual error still exceeds the threshold, the solution with the smallest residual error is taken as the optimal positioning result for the target after removing multipath signals from all base stations and considering only valid signals in the angle calculation.

**Figure 11:**
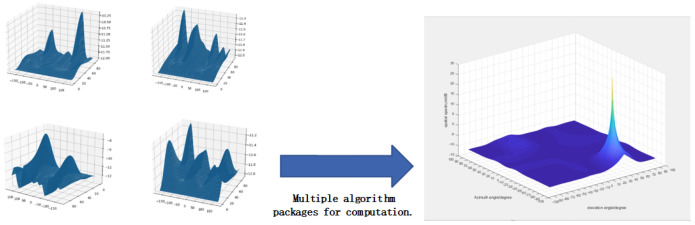
Simulation of improved algorithm estimation.

Based on the fused least squares and MUSIC positioning algorithm, combined with the anti-multipath algorithm, the angle information of the target to be measured can be effectively calculated. This will ultimately make the mapped coordinates more accurate, as shown in Figure 11. Combining the estimation of the multipath algorithm, after multiple optimization calculations, forms the optimal target estimation result.

## 5. Bluetooth AOA/AOD Indoor Localization Algorithm Testing

**Figure 12:**
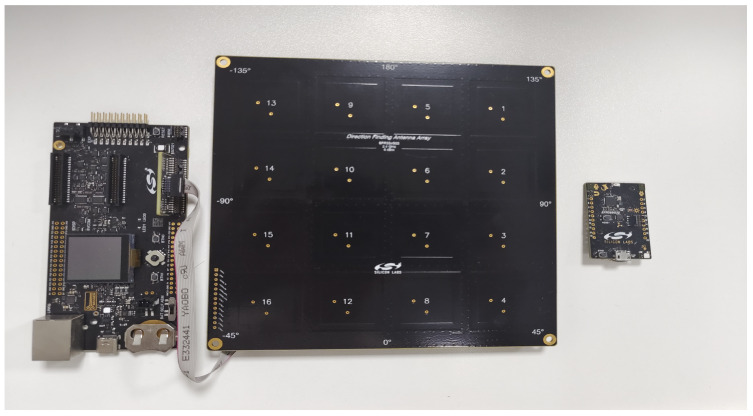
Test verification hardware.

The test verification hardware adopts a 4 × 4 dual-polarized antenna array based on Bluetooth 5.1 and corresponding electronic tags for the actual testing of the algorithms designed in this study, as shown in Figure 12. Compared to other antennas, the dual-polarized array antenna has better anti-interference capabilities and can improve spectrum utilization in spatial reuse, reducing the complexity of communication systems. It also offers higher flexibility in the configuration for different scenarios.

### Indoor Experimental Scene and Test Results

**Figure 13:**
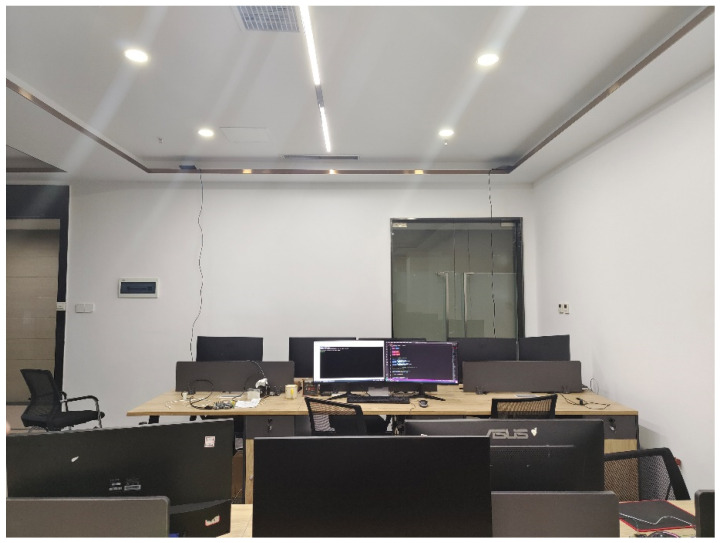
Indoor testing environment.

**Figure 14:**
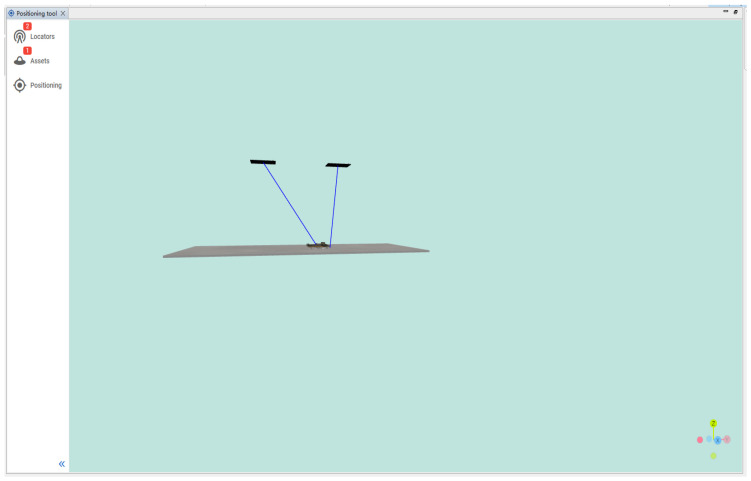
Three-dimensional proportional testing display.

In a complex indoor environment, the hardware antenna kit selected for verification is used to deploy two antenna array boards at the height of the ceiling. The first one is deployed on the left side of the corner of the ceiling, and the other one is deployed on the right side. The two are 1.5 m apart and 3 m away from the ground, as shown in Figure 13. The antenna arrays are connected to the switch of the same route through a network cable, and the collected data can be sent to the PC server of the same network segment for the algorithm solution and testing and other applications. By solving the collected signals into corresponding angles, the angle information is mapped to the simulation diagram coordinates in combination with the simulation software to form a real-time display of the test target. As shown in Figure 14, according to the actual test hardware deployment, the proportional simulation mapping is performed on the test platform, and the test status and the relative position of the positioning target can be displayed in real time in 3D. According to the debugging platform, it can be intuitively seen that the combination of algorithm and hardware verification has a good effect, and the algorithm can accurately obtain the target position status in target positioning.

**Figure 15:**
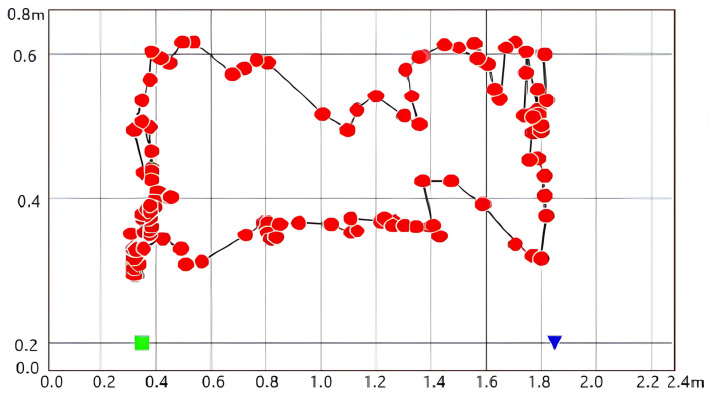
Display of 2D proportional testing trajectory.

**Figure 16:**
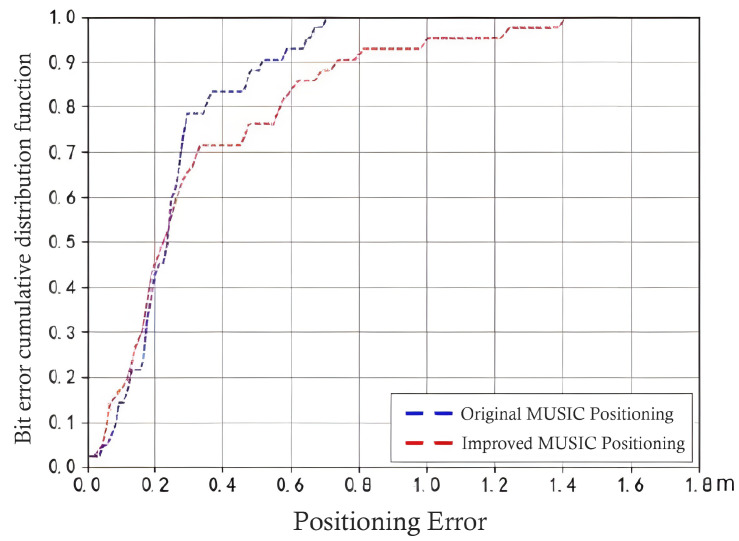
Comparison test of algorithm’s localization error.

According to the good results obtained by the 3D simulation test of the platform, a continuous test of multiple positions was further carried out indoors. The target coordinates obtained in each test were marked in the 2D proportional view, and connected in sequence according to the order of the trajectory coordinates to form complete moving target trajectory tracking. As shown in Figure 15, the test process moves in a rectangular trajectory, and it can be intuitively seen in the 2D view that most of the coordinates obtained in the test are presented on the rectangular trajectory, but a small number of coordinates still deviate from the correct position. According to all the tests, it can be seen that the algorithm has a high-precision positioning effect at the sub-meter level, and has a good ability to overcome the indoor multipath effect. Regardless of whether the data structure obtained by AOA or AOD is exactly the same, the algorithm also meets different scene applications in terms of universality, as shown in Figure 16. Finally, the overall test comparison of the algorithm in positioning and the algorithm without improved design is carried out within a certain range. The same test method as in Table 1 is used, just replacing the simulation signal with the actual collected real signal for testing. Other parameters remain unchanged. Finally, the positioning error data are recorded. The test data show that it is obvious that the improved algorithm has a large improvement in overall error and presents a good solution effect on the indoor multipath effect.

## 6. Conclusions and Prospects

Based on Bluetooth 5.1 AOA and AOD indoor positioning, as various hardware manufacturing technologies advance, indoor positioning research has shifted its core focus more towards algorithm research and design. Facing different scenarios and applications, hardware technology has already reached the current highest level, while there is still ample room for improvement and refinement in algorithm implementation. This paper starts from the perspective of the newly added direction-finding feature of Bluetooth 5.1, conducting research on its principles, implementation processes, and applications, to provide sufficient theoretical support for subsequent research and directions. Then, based on this research foundation, further exploration is conducted into the implementation of positioning algorithms to form an optimal preliminary algorithm model. Finally, starting from practical problems, the initial algorithm model is upgraded and improved to meet the core requirements of current positioning, achieving algorithm enhancement in terms of accuracy and environmental problem solving.

In the research and testing comparison of various algorithms, this paper selects the MUSIC algorithm, which demonstrates relatively superior performance in all aspects, for design. Moreover, in the final practical experimental tests, it exhibits significant improvement. The achievements and processes of this study hold high reference and learning value in the current field of indoor positioning, providing valuable insights for rapid validation of positioning solutions and confirmation of research directions. In the future development and application, indoor positioning will undoubtedly be an indispensable hotspot, contributing more application value in the era of big data and IoT. The research and implementation of core positioning algorithms will ultimately become the key competitive advantage in indoor positioning.

## Figures and Tables

**Table 1:** ESPRIT simulation parameter record.

Antennas	SNR	Sampling	Angle	Accuracy	Angle	Accuracy	Time
4 × 4	10 dB	1000	30°	95%	10°	95%	5 ms
4 × 4	10 dB	1000	60°	85%	30°	91%	4 ms
4 × 4	10 dB	1000	90°	90%	50°	93%	5 ms
4 × 4	10 dB	1000	120°	90%	70°	94%	3 ms
4 × 4	10 dB	1000	150°	95%	90°	96%	4 ms

**Table 2:** Capon simulation parameter record.

Antennas	SNR	Sampling	Angle	Accuracy	Angle	Accuracy	Time
4 × 4	10 dB	1000	30°	90%	10°	91%	8 ms
4 × 4	10 dB	1000	60°	80%	30°	88%	6 ms
4 × 4	10 dB	1000	90°	85%	50°	92%	7 ms
4 × 4	10 dB	1000	120°	90%	70°	85%	5 ms
4 × 4	10 dB	1000	150°	83%	90°	95%	4 ms

**Table 3:** MUSIC simulation parameter record.

Antennas	SNR	Sampling	Angle	Accuracy	Angle	Accuracy	Time
4 × 4	10 dB	1000	30°	93%	10°	96%	8 ms
4 × 4	10 dB	1000	60°	90%	30°	95%	6 ms
4 × 4	10 dB	1000	90°	89%	50°	94%	7 ms
4 × 4	10 dB	1000	120°	94%	70°	95%	5 ms
4 × 4	10 dB	1000	150°	86%	90°	97%	4 ms

## Data Availability

Data are contained within the article.
